# Book review for “Orthognathic Surgery: Principles, Planning and Practice”

**DOI:** 10.1186/s40902-017-0119-z

**Published:** 2017-06-25

**Authors:** Seong-Gon Kim

**Affiliations:** Department of Oral and Maxillofacial Surgery, Gangneung-Wonju National University, Gangneung, Republic of Korea

## Editorial

Recently, my close friend, Farhad Naini (London, UK), published a textbook entitled “Orthognathic Surgery: Principles, Planning and Practice” [1]. Though there have been many textbooks for orthognathic surgery, this textbook is unique. There are 61 chapters and 95 contributing authors. The book begins with a wonderful introductory chapter on the historical background of orthognathic surgery written by the father of modern orthognathic surgery, Prof. Hugo Obwegeser. As Dr Naini is an orthodontist, he has included many chapters on pre-operative and post-operative orthodontic consideration for orthognathic surgery. The details about surgical technique are also covered in detail. This book should be added to the catalogue of must-read books for both oral and maxillofacial surgeons and orthodontists. In addition, Dr Naini has published very interesting articles in Maxillofacial Plastic and Reconstructive Surgery [2, 3]. As Maxillofacial Plastic and Reconstructive Surgery is an open access journal, you can read Dr Naini’s recent works for free.
